# Involvement of Type 1 Angiontensin II Receptor (AT_1_) in Cardiovascular Changes Induced by Chronic Emotional Stress: Comparison between Homotypic and Heterotypic Stressors

**DOI:** 10.3389/fphar.2016.00262

**Published:** 2016-08-18

**Authors:** Willian Costa-Ferreira, Jonas O. Vieira, Jeferson Almeida, Lucas Gomes-de-Souza, Carlos C. Crestani

**Affiliations:** ^1^Faculdade de Ciências Farmacêuticas, UNESP-Universidade Estadual PaulistaAraraquara, Brazil; ^2^Joint UFSCar-UNESP Graduate Program in Physiological Sciences, UFSCar-UNESPSão Carlos, Brazil

**Keywords:** losartan, cardiovascular, baroreflex, autonomic, restraint stress, chronic variable stress

## Abstract

Consistent evidence has shown an important role of emotional stress in pathogenesis of cardiovascular diseases. Additionally, studies in animal models have demonstrated that daily exposure to different stressor (heterotypic stressor) evokes more severe changes than those resulting from repeated exposure to the same aversive stimulus (homotypic stressor), possibly due to the habituation process upon repeated exposure to the same stressor. Despite these pieces of evidence, the mechanisms involved in the stress-evoked cardiovascular dysfunction are poorly understood. Therefore, the present study investigated the involvement of angiotensin II (Ang II) acting on the type 1 Ang II receptor (AT_1_) in the cardiovascular dysfunctions evoked by both homotypic and heterotypic chronic emotional stresses in rats. For this purpose, we compared the effect of the chronic treatment with the AT_1_ receptor antagonist losartan (30 mg/kg/day, p.o.) on the cardiovascular and autonomic changes evoked by the heterotypic stressor chronic variable stress (CVS) and the homotypic stressor repeated restraint stress (RRS). RRS increased the sympathetic tone to the heart and decreased the cardiac parasympathetic activity, whereas CVS decreased the cardiac parasympathetic activity. Additionally, both stressors impaired the baroreflex function. Alterations in the autonomic activity and the baroreflex impairment were inhibited by losartan treatment. Additionally, CVS reduced the body weight and increased the circulating corticosterone; however, these effects were not affected by losartan. In conclusion, these findings indicate the involvement of angiotensin II/AT_1_ receptors in the autonomic changes evoked by both homotypic and heterotypic chronic stressors. Moreover, the present results provide evidence that the increase in the circulating corticosterone and body weight reduction evoked by heterotypic stressors are independent of AT_1_ receptors.

## Introduction

Consistent evidence from animals and humans studies has shown an important role of emotional stress in the pathogenesis of cardiovascular diseases (Kivimäki et al., 2006; Jarczok et al., 2013). Preclinical studies reported mild hypertension, elevated resting heart rate (HR), cardiac hypertrophy and contractile dysfunction, and increased susceptibility to arrhythmias in rodents exposed to chronic stressors (Grippo et al., 2004, 2008; Duarte et al., 2015). These responses were followed by autonomic imbalance and impairment of the baroreflex function (Conti et al., 2001; Grippo et al., 2008; Bouzinova et al., 2012; Almeida et al., 2015; Duarte et al., 2015). Additionally, *in vitro* and *in vivo* studies identified changes in the vascular function due to stressor (Neves et al., 2009; Isingrini et al., 2012; Baptista et al., 2014; Duarte et al., 2015).

The predictability of the aversive stimuli applied repeatedly has been proposed to be an important factor determining its consequences (Herman, 2013; Crestani, 2016). The influence of stressor predictability has been evaluated in animal models by comparing the effect of chronic stressors involving daily exposure to the same type of stressor (i.e., homotypic/predictable) versus different aversive stimuli (i.e., heterotypic/unpredictable; Crestani, 2016). Typically, the chronic variable stress (CVS) has been employed as a heterotypic stressor and the repeated restraint stress (RRS) as a homotypic stressor (Crestani, 2016). Studies comparing RRS and CVS have demonstrated that the latter exhibits a more severe impact on the somatic parameters (e.g., adrenal hypertrophy and thymic involution), hypothalamic-pituitary-adrenal (HPA) axis activity, and anxiety- and depression-like behaviors (Magarinos and McEwen, 1995; Haile et al., 2001; Marin et al., 2007; Pastor-Ciurana et al., 2014). Differences in the cardiovascular and autonomic changes following exposure to predictable versus unpredictable stressors were addressed only recently. Indeed, Duarte et al. (2015) reported that increase in the cardiac sympathetic activity, resting tachycardia, and baroreflex impairment was only caused by CVS, although both stressors caused mild hypertension. Altogether, these pieces of evidence indicate a more severe impact of CVS compared to RRS on the neuroendocrine, behavioral, somatic, and cardiovascular responses to stress. The less severe impact of RRS is possibly related to a habituation process of the physiological responses upon repeated exposure to the same stressor, which minimize the impact of stress (Herman, 2013; Crestani, 2016; McCarty, 2016). Additionally, RRS and CVS differently affect the morphology and function of the limbic structures in the brain (Flak et al., 2012; Kopp et al., 2013; Smith et al., 2016).

Angiotensin II (Ang II) is an active peptide of the renin-angiotensin system (RAS) that plays a key role in the control of the cardiovascular function and hydroelectrolytic homeostasis (Ménard, 1993; Head, 1996). The effects of Ang II are mediated via activation of two receptors, denominated type 1 Ang II (AT_1_) and type 2 Ang II (AT_2_) receptors (de Gasparo et al., 2000; Karnik et al., 2015). In addition to its synthesis and action in the circulation as a blood-borne hormone, Ang II is also synthetized locally in various tissues, including the brain (McKinley et al., 2003; Wright and Harding, 2011). Relevant to stress is the identification of the RAS components and Ang II receptors in the brain areas controlling the stress responses (Wright and Harding, 2011; Bali and Jaggi, 2013).

Previous studies demonstrated that systemic or intra cerebroventricular administration of selective AT_1_ receptor antagonists decreased the cardiovascular responses observed during acute sessions of stress (Saiki et al., 1997; Jezova et al., 1998; Kubo et al., 2001; Erdos et al., 2010; Busnardo et al., 2014). However, a possible role of the angiontensinergic mechanisms in the cardiovascular dysfunctions evoked by chronic stress has never been investigated. Nevertheless, an increase in Ang II levels in the plasma and brain tissue as well as in the expression of AT_1_ receptors in the brain has been reported following exposure to chronic stressors (Castren and Saavedra, 1988; Yang et al., 1994; McDougall et al., 2000; Chung et al., 2010). Additionally, systemic treatment with AT_1_ receptor antagonists inhibited the endocrine changes, hyperglycemia, and vascular oxidative stress induced by chronic stress (Uresin et al., 2004; Chung et al., 2010). These findings support the hypothesis of the involvement of Ang II/AT_1_ receptor in the autonomic/cardiovascular changes evoked by chronic stress. Therefore, the purpose of the present study was to investigate the involvement of Ang II activating the AT_1_ receptor in the cardiovascular and autonomic changes caused by chronic emotional stress. Additionally, this study aimed to provide further evidence regarding the influence of the predictability of stressor stimulus on the resulting cardiovascular and autonomic changes. Therefore, we compared the effect of treatment with the selective AT_1_ receptor antagonist losartan on the autonomic/cardiovascular changes evoked by the heterotypic stressor CVS and the homotypic stressor RRS.

## Materials and Methods

### Animals

Forty-five male Wistar rats (200 g) were obtained from the animal breeding facility of the São Paulo State University–UNESP (Botucatu, SP, Brazil). Animals were housed in collective plastic cages in a temperature-controlled room at 24°C at the Animal Facility of the Laboratory of Pharmacology, School of Pharmaceutical Sciences, São Paulo State University–UNESP. They were kept under a 12:12 h light-dark cycle (lights on between 7:00 a.m. and 7:00 p.m.) with free access to water and standard laboratory food. The housing conditions and experimental procedures were carried out following the protocols approved by the local Ethical Committee for Use of Animal at the School of Pharmaceutical Sciences-UNESP (approval number: 32/2014), which complies with the Brazilian and international guidelines for animal use and welfare.

### Chronic Stress Regimens

The protocols of chronic stress were based on previous studies of our group (Marin et al., 2007; Duarte et al., 2015). Therefore, RRS was chosen as a homotypic stressor, whereas CVS was used as a heterotypic stress regimen. The animals in the RRS groups were restrained in opaque plastic cylinders (15 cm length and 5.5 cm internal diameter) for 1 h daily starting at 9:00 a.m. for 10 consecutive days. The CVS protocol involved the exposure to different stressors in a variable schedule for 10 consecutive days, according to the protocol previously described by our group (Marin et al., 2007; Duarte et al., 2015). The stressors used in the CVS protocol included: (1) restraint stress (60 min); (2) humid sawdust (overnight or all day); (3) cold (4°C)/room temperature isolation housing; (4) food/water deprivation; (5) swim stress (4 min); (6) lights on overnight; and (7) lights off during day (120–180 min). All stress sessions were performed in an adjacent room to the animal facility. RRS and CVS started simultaneously, and during this period, animals of the control groups were left undisturbed, except for cleaning the cages and pharmacological treatment, in the animal facility.

### Pharmacological Treatment

The pharmacological treatment with losartan (selective AT_1_ receptor antagonist) started on the first day of the stress protocols and was continued daily for 10 consecutive days. Losartan was given daily by gavage at a dose of 30 mg/kg/day (Uresin et al., 2004).

### Surgical Preparation

At the 10th day of the stress protocols, after the last stress session, all animals were anesthetized with tribromoethanol (250 mg/kg, i.p.) and a catheter (a 4-cm segment of PE-10 heat-bound to a 13 cm segment of PE-50; Clay Adams, Parsippany, NJ, USA) was inserted into the abdominal aorta through the femoral artery for arterial pressure recording. A second catheter was implanted into the femoral vein for infusion of drugs. Both catheters were tunneled under the skin and exteriorized on the animal’s dorsum. After the surgery, rats were treated with a polyantibiotic formulation of streptomycin and penicillin (560 mg/mL/kg, i.m.) to prevent infection, and received the non-steroidal anti-inflammatory drug flunixin meglumine (0.5 mg/mL/kg, s.c.) for postoperative analgesia.

### Measurement of Cardiovascular Parameters

The arterial cannula was connected to a pressure transducer (DPT100, Utah Medical Products Inc., Midvale, UT, USA). The pulsatile arterial pressure was recorded using an amplifier (Quad Bridge Amp, ML224, ADInstruments, Sydney, NSW, Australia) and an acquisition board (PowerLab 4/30, ML866/P, ADInstruments, Sydney, NSW, Australia) connected to a personal computer. The mean arterial pressure (MAP), systolic arterial pressure (SAP), diastolic arterial pressure (DAP), and HR values were derived from the pulsatile arterial pressure values.

### Assessment of the Autonomic Activity and Intrinsic HR

The cardiac autonomic tone and intrinsic HR (iHR) were assessed via the intravenous administration of methylatropine (muscarinic receptor antagonist; 3 mg/ml/kg) and propranolol (β-adrenoceptor antagonist; 4 mg/ml/kg). The protocol was performed on 2 days. On the first day, the rats in all the experimental groups received intravenous methylatropine and propranolol in a random order. The interval between the drug treatments was 10 min. On the subsequent day, the rats were treated with methylatropine and propranolol in the opposite sequence to that used on the first day. The parasympathetic activity was determined from the change in the basal HR caused by methylatropine, whereas the sympathetic activity was determined from the change in the HR following propranolol treatment. The iHR was determined after the combined treatment with propranolol and methylatropine on the first and second days of the experiment, and a mean value was calculated for each animal.

The power spectral analysis of SAP was used to analyze the sympathetic activity controlling the vascular tone. For this purpose, the beat-to-beat time series of SAP were extracted from the pulsatile arterial pressure signal. Using the Cardioseries v2.4 software^1^, the overall variability of these series was calculated in the time and frequency domain, and the power of the obtained oscillatory components was quantified in frequency bands of 0.20–0.75 Hz (low frequency, LF). The oscillations of arterial pressure at LF range are representative of the modulatory effects of the sympathetic system controlling the vascular tone (Malliani et al., 1991; Janssen et al., 2000; Just et al., 2000; Ramaekers et al., 2002).

### Infusion of Vasoactive Agents

Intravenous infusions of the selective α_1_-adrenoceptor agonist phenylephrine (70 μg/mL at 0.4 mL/min/kg), the nitric oxide donor sodium nitroprusside (SNP; 100 μg/mL at 0.8 mL/min/kg), and acetylcholine (10 μg/mL at 1.2 mL/min/kg) was administered using an infusion pump (KD Scientific, Holliston, MA, USA) (Almeida et al., 2015; Duarte et al., 2015). The infusions of the vasoactive drugs were randomized, and the second treatment was not given before the cardiovascular parameters returned to the control values (the interval between the infusions was approximately 5 min). The infusions lasted for 20–30 s, resulting in the administration of a total dose of 9–14 μg/kg of phenylephrine, 26–40 μg/kg of SNP, and 4–6 μg/kg of acetylcholine.

### Assessment of the Baroreflex Activity

Analysis of the baroreflex activity was carried out using two methods: (i) the classical pharmacological approach, and (ii) the sequence analysis technique. For the classical pharmacological analysis, curves of the MAP variations (10, 20, 30, and 40 mmHg) evoked by phenylephrine and SNP infusions versus the reflex HR responses were constructed. Paired values of MAP and HR changes were plotted to generate sigmoid logistic functions, which were used to determine the baroreflex activity (Head and McCarty, 1987; Crestani et al., 2010a). The baroreflex analysis using the sigmoid curves was characterized by five parameters: (i) lower HR plateau (P_1_, bpm) (i.e., the maximum reflex bradycardia); (ii) upper HR plateau (P_2_, bpm) (i.e., the maximum reflex tachycardia); (iii) HR range (bpm) (i.e., the difference between the upper and lower plateau levels); (iv) median blood pressure (BP_50_, mmHg), which is the MAP at 50% of the HR range; and (v) average gain (G, bpm/mmHg), which is the average slope of the curves between +1 and -1 standard derivations from the BP_50_ (Head and McCarty, 1987; Crestani et al., 2010a). To analyze the reflex responses during pressor and depressor effects separately, the HR values matching MAP changes were plotted to create linear regression curves and their slopes were compared to evaluate the changes in the baroreflex gain (Crestani et al., 2010b, 2011; Almeida et al., 2015).

The sequence method was used to evaluate the baroreflex function over the physiological range of fluctuations in the arterial pressure, without any pharmacological manipulation. For this purpose, the beat-to-beat values of SAP and PI were analyzed using the software Cardioseries v2.4 (Granjeiro et al., 2014; Almeida et al., 2015) for identification of the sequences in which the SAP increase was associated with PI lengthening (up sequence) or the SAP decrease was associated with PI shortening (down sequence). A baroreflex sequence was only used when the correlation coefficient (*r*) between the SAP and PI was greater than 0.8. The spontaneous baroreflex sensitivity was assessed based on the slope (ms/mmHg) of the linear regression between the SAP and PI.

### Vascular Reactivity to the Vasoactive Agents

The graded changes in the MAP evoked by the intravenous infusion of the pressor agent phenylephrine and the depressor agents SNP and acetylcholine were plotted to generate dose–response curves (Crestani et al., 2011; Almeida et al., 2015; Duarte et al., 2015). Dose–response curves were constructed for each vasoactive agent by calculating the amount of drug infused and the MAP change every 2 s after starting the infusion. The maximal effect (*E*_max_) and the dose at 50% of the MAP range (ED_50_) for each vasoactive agent were compared in all experimental groups.

### Plasma Corticosterone Measurements

Blood sample (200 μL) was collected from the femoral artery catheter of each animal for determination of plasma corticosterone concentration. Blood was collected in plastic tubes containing 5 μL of heparin (5000 UI/mL). Samples were centrifuged at 2000 × *g* for 10 min at 4°C and plasma was stored at -20°C until the corticosterone assay was carried out.

Plasma corticosterone concentration was measured by radioimmunoassay. The method was adapted from that described previously (Sarnyai et al., 1992). Briefly, 20 μL of plasma was diluted 50 times with 0.01 M phosphate-buffered saline (PBS) and placed in a water bath at 75°C for 1 h for heat inactivation of corticosteroid binding globulin. One-hundred microliters of a solution of antibody (Sigma–Aldrich, St. Louis, MO, USA) and (3H)-corticosterone (New England Nuclear, Boston, MA, USA; 10,000–20,000 cpm/mL) was added to each sample, mixed and incubated overnight at 4°C. Dextran-coated charcoal was used to adsorb the free steroid after incubation. The tubes were centrifuged at 2000 × *g* for 15 min at 4°C, the supernatant from each tube was transferred to scintillation vials and the radioactivity was quantified by liquid scintillation spectrometry. Standard curves were constructed using 25, 50, 100, 250, 500, 750, 1000, and 2000 pg/100 μL of corticosterone (Sigma–Aldrich, St. Louis, MO, USA). After dilution, all the concentrations of corticosterone samples were within the linear range of the standard curve.

### Drugs and Solutions

Losartan (Sigma–Aldrich, St Louis, MO, USA), propranolol hydrochloride (Sigma–Aldrich), methylatropine (Sigma–Aldrich), phenylephrine hydrochloride (Sigma–Aldrich), sodium nitroprusside (Sigma–Aldrich), acetylcholine (Sigma–Aldrich), tribromoethanol (Sigma–Aldrich), and urethane (Sigma–Aldrich) were dissolved in saline (0.9% NaCl). Flunixin meglumine (Banamine^®^; Schering-Plough, Cotia, SP, Brazil) and the poly-antibiotic preparation (Pentabiotico^®^; Fort Dodge, Campinas, SP, Brazil) were used as provided.

### Experimental Procedures

The rats were divided into six groups: (i) control vehicle (*n* = 6), (ii) control losartan (*n* = 8), (iii) RRS vehicle (*n* = 8), (iv) RRS losartan (*n* = 8), (v) CVS vehicle (*n* = 8), and (vi) CVS losartan (*n* = 7). The protocols of chronic stress and the pharmacological treatment with losartan were started on the same day and continued for 10 consecutive days. At the 10th day, after the last session of stress/treatment, animals in all experimental groups were subjected to surgical preparation. Since the purpose of the present study was to investigate the enduring cardiovascular and autonomic changes evoked by chronic stress exposure, the tests were performed on days 11 and 12 after the onset of chronic stress protocols (i.e., 24 and 48 h after the last stress session).

On the testing days, animals were transferred to the experimental room in their home box. They were allowed to adapt to the experimental room conditions, such as sound and illumination, for 60 min before starting the experiments. The experimental room was temperature controlled (24°C) and was acoustically isolated from the other rooms. On the morning of the first testing day, a blood sample (200 μL) was collected from the femoral artery catheter of each animal for determination of plasma corticosterone concentration. In the sequence, animals were subjected to a 30-min period of basal recording of the arterial pressure and HR. After that, they received intravenous infusions of phenylephrine (selective α_1_-adrenoceptor agonist), SNP (nitric oxide donor) and acetylcholine in a random order. After the infusion of the vasoactive agents, animals in all experimental groups received intravenous methylatropine and propranolol in a random order. The interval between the administrations of the autonomic blockers was 10 min. On the second testing day, animals were treated with methylatropine and propranolol in an opposite sequence to that used on day 1. Treatment was carried out following the same procedure (10-min interval) described on the day 1. At the end of the experiments, the rats were euthanized via anesthetic overdose (urethane, 250 mg/mL/200 g body weight, i.p.) and the heart, adrenals, and thymus were removed and weighed.

### Data Analysis

Data were expressed as the mean ± SEM. The values of body weight were analyzed using three-way ANOVA followed by Bonferroni *post hoc* test, with stress (control, RRS, and CVS) and treatment (vehicle and losartan) as independent factors and time as repeated measurement. Other measurements were analyzed using two-way ANOVA followed by Bonferroni *post hoc* test, with stress and treatment as independent factors. Results of statistical tests with *P* < 0.05 were considered significant.

## Results

### Effects of Chronic Stress and Losartan Treatment on the Somatic Parameters and Plasma Corticosterone Concentration

**FIGURE 1:**
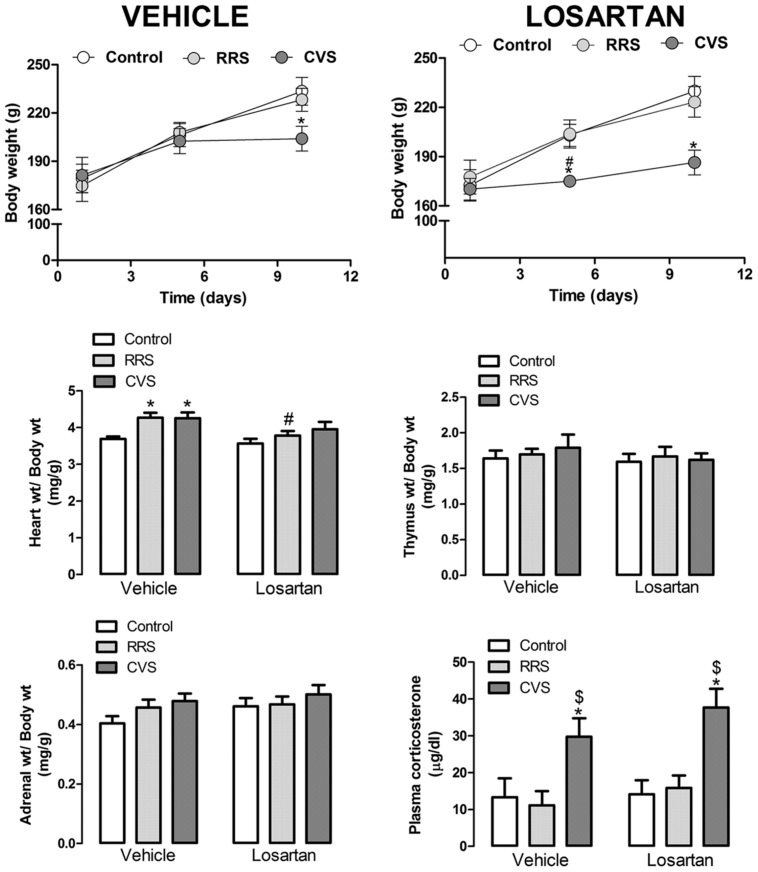
**Body, heart, thymus, adrenal, and kidneys weights; and plasma corticosterone concentration in animals treated with vehicle or losartan control (white symbols) and subjected to repeated restraint stress (RRS; light gray symbols) or chronic variable stress (CVS; dark gray symbols).** **(Top)** Time course curve of body weight. The data are mean ± SEM. ^∗^*p* < 0.05 versus respective control group within same condition, ^#^*p* < 0.05 versus respective vehicle group. Three-way ANOVA followed by Bonferroni *post hoc* test. **(Bottom)** Relative weight (weight/body weight) of heart, thymus, and adrenal; and plasma corticosterone concentration. The bars represent the mean ± SEM. ^∗^*p* < 0.05 versus control group within same condition, ^#^*p* < 0.05 versus respective vehicle group, ^$^*p* < 0.05 CVS versus RRS within same condition. Two-way ANOVA followed by Bonferroni *post hoc* test. (control vehicle: *n* = 6, control losartan: *n* = 8, RRS vehicle: *n* = 8, RRS losartan: *n* = 8, CVS vehicle: *n* = 8, CVS losartan: *n* = 7).

Analysis of body weight gain indicated a main effect of time [*F*_(2,96)_ = 202.03, *P* < 0.001] and stress × time interaction [*F*_(4,96)_ = 12.73, *P* < 0.001]; however, the stress [*F*_(2,48)_ = 1.24, *P* > 0.05] and pharmacological treatment [*F*_(1,48)_ = 0.96, *P* > 0.05] had not effect. Treatment × time [*F*_(2,96)_ = 0.52, *P* > 0.05], stress × treatment [*F*_(2,48)_ = 0.04, *P* > 0.05], and stress × treatment × time [*F*_(4,96)_ = 0.64, *P* > 0.05] interactions also did not reach significance (**Figure 1**). The *post hoc* analysis revealed that CVS reduced the body weight in the vehicle-treated animals at day 10 (*P* < 0.01), as well as in the losartan-treated rats at days 5 (*P* < 0.05) and 10 (*P* < 0.01). RRS did not affect the body weight gain (*P* > 0.05; **Figure 1**).

Analysis of the relative heart weight (heart weight/body weight) indicated a major effect of stress [*F*_(2,39)_ = 6.13, *P* < 0.004] and treatment [*F*_(1,39)_ = 6.70, *P* < 0.01], without stress × treatment interaction [*F*_(1,39)_ = 0.77, *P* > 0.05] (**Figure 1**). The *post hoc* analysis revealed that RRS and CVS increased the relative heart weight (*P* < 0.05); however, this effect was not identified in the losartan-treated animals (*P* > 0.05; **Figure 1**). Comparison of the relative weights of the thymus and adrenals did not indicate an effect of either stress [thymus: *F*_(2,39)_ = 0.28, *P* > 0.05; adrenals: *F*_(2,39)_ = 2.18, *P* > 0.05] or treatment [thymus: *F*_(1,39)_ = 0.65, *P* > 0.05; adrenals: *F*_(1,39)_ = 1.89, *P* > 0.05] (**Figure 1**).

Analysis of the plasma corticosterone concentration indicated an effect of stress [*F*_(2,39)_ = 13.96, *P* < 0.0001]; however, the treatment [*F*_(1,39)_ = 1.51, *P* > 0.05] and stress × treatment interaction [*F*_(2,39)_ = 0.32, *P* > 0.05] showed no effect (**Figure 1**). The *post hoc* analysis revealed that CVS increased the corticosterone concentration (*P* < 0.05); however, losartan treatment did not affect this effect (*P* < 0.05). RRS did not affect the plasma corticosterone levels (*P* > 0.05; **Figure 1**).

### Effects of Chronic Stress and Losartan Treatment on the Basal Cardiovascular Parameters

**FIGURE 2:**
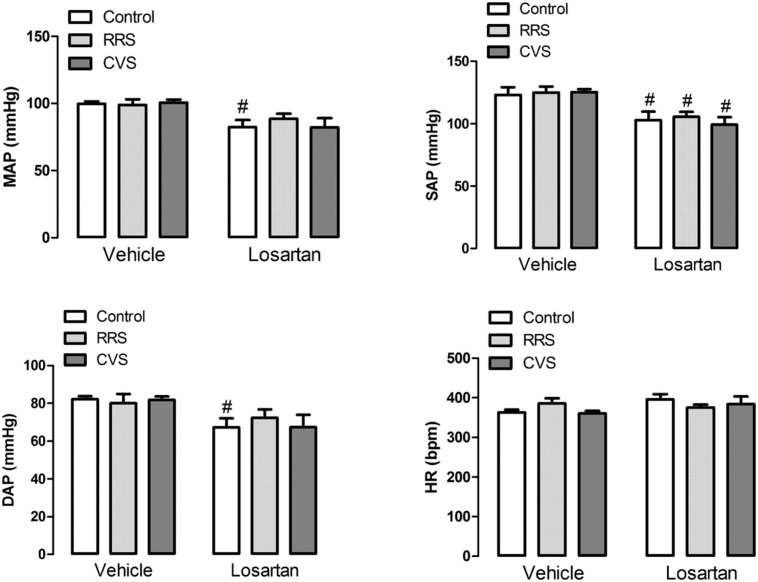
**Mean (MAP), systolic (SAP), and diastolic (DAP) arterial pressure; and heart rate (HR) in animals treated with vehicle or losartan control (white bars) and subjected to RRS (light gray bars) or CVS (dark gray bars).** The bars represent the mean ± SEM. ^#^*p* < 0.05 versus respective vehicle group. Two-way ANOVA followed by Bonferroni *post hoc* test. (control vehicle: *n* = 6, control losartan: *n* = 8, RRS vehicle: *n* = 8, RRS losartan: *n* = 8, CVS vehicle: *n* = 8, CVS losartan: *n* = 7).

Analysis of the basal values of HR did not indicate an effect of either stress [*F*_(2,39)_ = 0.27, *P* > 0.05] or treatment [*F*_(1,39)_ = 2.12, *P* > 0.05] (**Figure 2**). However, analysis of both MAP, SAP, and DAP indicated an effect of losartan treatment [MAP: *F*_(1,39)_ = 16.71, *P* < 0.05; SAP: *F*_(1,39)_ = 25.97, *P* < 0.05; DAP: *F*_(1,39)_ = 11.07, *P* < 0.05], but without effect of stress [MAP: *F*_(2,39)_ = 0.19, *P* > 0.05; SAP: *F*_(2,39)_ = 0.17, *P* > 0.05; DAP: *F*_(2,39)_ = 0.09, *P* > 0.05] and stress × treatment interaction [MAP: *F*_(2,39)_ = 0.48, *P* > 0.05; SAP: *F*_(2,39)_ = 0.24, *P* > 0.05; DAP: *F*_(2,39)_ = 0.39, *P* > 0.05] (**Figure 2**). The *post hoc* analysis revealed that losartan reduced SAP in all experimental groups (*P* < 0.05), whereas MAP (*P* < 0.05) and DAP (*P* < 0.05) were reduced only in the control group (**Figure 2**).

### Effects of Chronic Stress and Losartan Treatment on the Autonomic Activity and Intrinsic HR

#### Cardiac Sympathetic Activity

**FIGURE 3:**
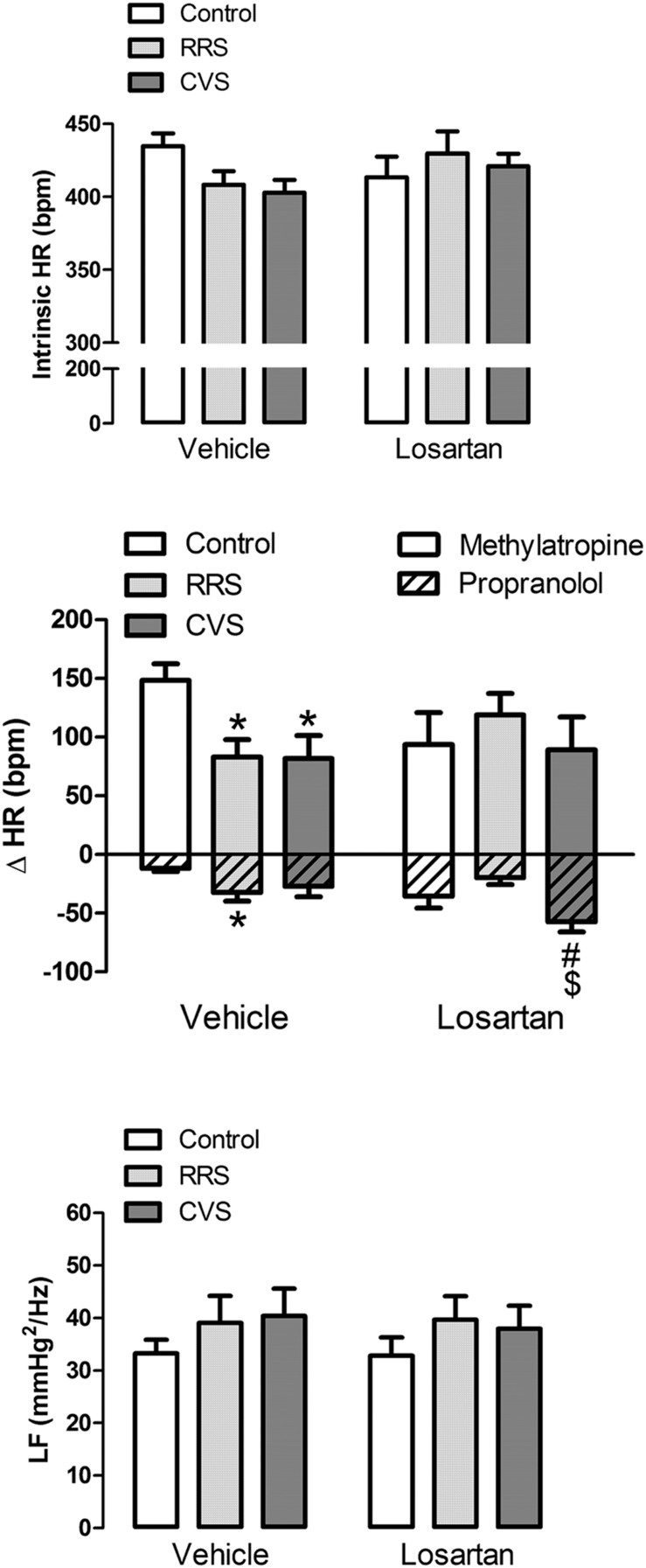
**Intrinsic HR (iHR) and autonomic activity in animals treated with vehicle or losartan control (white bars) and subjected to RRS (light gray bars) or CVS (dark gray bars).** **(Top)** HR values after combined treatment with propranolol and methylatropine (iHR). **(Middle)** HR changes (ΔHR) evoked by administration of methylatropine (positive values, open bars) or propranolol (negative values, cross-hatched bars). **(Bottom)** Power spectral analysis of systolic arterial pressure (SAP). Graphs present the oscillation at low frequency (LF) range. The bars represent the mean ± SEM. ^∗^*p* < 0.05 versus control group within same condition. ^#^*p* < 0.05 versus respective vehicle group. ^$^*p* < 0.05 CVS versus RRS within same condition. Two-way ANOVA followed by Bonferroni *post hoc* test. (control vehicle: *n* = 6, control losartan: *n* = 8, RRS vehicle: *n* = 8, RRS losartan: *n* = 8, CVS vehicle: *n* = 8, CVS losartan: *n* = 7).

Analysis of the change in HR induced by intravenous administration of the β-adrenoceptor antagonist propranolol indicated a significant effect of stress [*F*_(2,39)_ = 3.24, *P* < 0.05] and treatment [*F*_(1,39)_ = 4.37, *P* < 0.05], as well as a treatment × stress interaction [*F*_(2,39)_ = 4.29, *P* < 0.05] (**Figure 3**). The *post hoc* analysis revealed that RRS increased propranolol response (*P* < 0.05), and this effect was inhibited by losartan treatment (*P* > 0.05; **Figure 3**). Furthermore, CVS increased propranolol-evoked HR change in the losartan-treated animals (*P* < 0.05; **Figure 3**).

#### Cardiac Parasympathetic Activity

Analysis of the change in HR induced by intravenous administration of the muscarinic cholinergic receptor antagonist methylatropine did not indicate an effect of either stress [*F*_(2,39)_ = 1.39, *P* > 0.05], losartan [*F*_(1,39)_ = 0.05, *P* > 0.05], or stress × treatment interaction [*F*_(2,39)_ = 2.40, *P* > 0.05] (**Figure 3**). However, the *post hoc* analysis revealed that both RRS (*P* < 0.05) and CVS (*P* < 0.05) reduced methylatropine response in the vehicle-treated animals, but not in the losartan-treated rats (*P* > 0.05; **Figure 3**).

#### Intrinsic HR

Analysis of the HR values after combined treatment with propranolol and methylatropine (iHR) did not indicate an effect of either stress [*F*_(2,39)_ = 0.57, *P* > 0.05] or treatment [*F*_(1,39)_ = 0.44, *P* > 0.05] (**Figure 3**).

#### Vascular Sympathetic Activity

Analysis of the oscillations of the SAP at LF ranges did not indicate an effect of either stress [*F*_(2,39)_ = 1.08, *P* > 0.05] or treatment [*F*_(1,31)_ = 0.04, *P* > 0.05] (**Figure 3**).

### Effects of Chronic Stress and Losartan Treatment on the Baroreflex Activity

**FIGURE 4:**
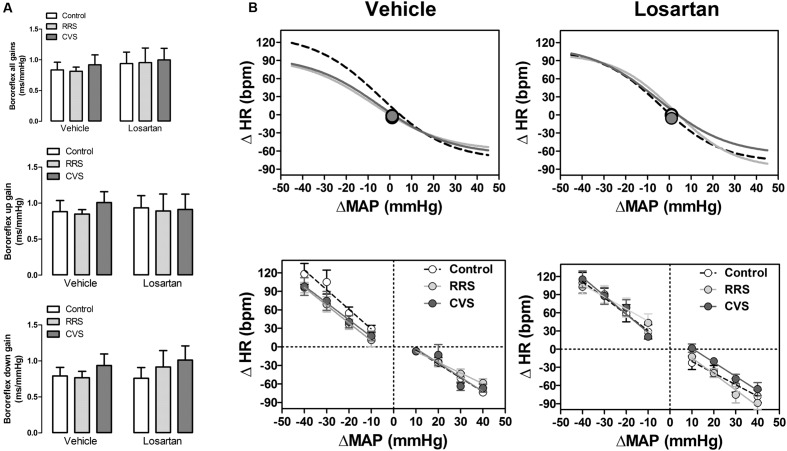
**Baroreflex activity in animals treated with vehicle or losartan control (white symbols) and subjected to RRS (light gray symbols) or CVS (dark gray symbols).** **(A)** Spontaneous baroreflex sensitivity (SBS) determined by the sequence analysis technique. Graphs present the mean of all slopes (baroreflex all gains, up and down sequence slopes; top) and the slopes of up (baroreflex up gain, middle) and down (baroreflex down gain, bottom) sequences. The bars represent the mean ± SEM. (control vehicle: *n* = 6, control losartan: *n* = 8, RRS vehicle: *n* = 8, RRS losartan: *n* = 8, CVS vehicle: *n* = 8, CVS losartan: *n* = 7). Two-way ANOVA followed by Bonferroni *post hoc* test. **(B)** Non-linear (top) and linear (bottom) regression analysis of the baroreflex correlating mean arterial pressure change (ΔMAP) evoked by intravenous infusion of phenylephrine and SNP and the reflex HR response (ΔHR). Symbols on sigmoid curves indicate the median blood pressure (BP_50_). (control vehicle: *n* = 6, control losartan: *n* = 8, RRS vehicle: *n* = 8, RRS losartan: *n* = 8, CVS vehicle: *n* = 8, CVS losartan: *n* = 7).

The baroreflex activity assessed by the sequence analysis technique did not indicate an effect of either stress [up: *F*_(2,39)_ = 0.14, *P* > 0.05; down: *F*_(2,39)_ = 0.70, *P* > 0.05; all: *F*_(2,39)_ = 0.12, *P* > 0.05] or treatment [up: *F*_(1,39)_ = 0.00, *P* > 0.05; down: *F*_(1,39)_ = 0.20, *P* > 0.05; all: *F*_(1,39)_ = 0.53, *P* > 0.05] regarding the slope of both the up and down sequences, as well as the mean of all gains (**Figure 4A**).

Results of the non-linear and linear analysis of the baroreflex activity are presented in **Figure 4B** and **Table 1.** The non-linear analysis of the baroreflex activity indicated an effect of stress [*F*_(2,39)_ = 5.01, *P* < 0.01] and treatment [*F*_(1,39)_ = 16.39, *P* < 0.0002], as well as a stress × treatment interaction [*F*_(2,39)_ = 3.80, *P* < 0.03] for the G parameter. Moreover, analysis of the HR range indicated a stress × treatment interaction [*F*_(2,39)_ = 3.30, *P* < 0.04], but without an effect of either stress [*F*_(2,39)_ = 2.30, *P* > 0.05] or treatment [*F*_(1,39)_ = 2.20, *P* > 0.05]. Analysis of P_1_, P_2_, and BP_50_ parameters did not indicate an effect of either stress [P_1_: *F*_(2,39)_ = 0.96, *P* > 0.05; P_2_: *F*_(2,39)_ = 2.78, *P* > 0.05; BP_50_: *F*_(2,39)_ = 0.55, *P* > 0.05] or treatment [P_1_: *F*_(1,39)_ = 3.22, *P* > 0.05; P_2_: *F*_(1,39)_ = 0.01, *P* > 0.05; BP_50_: *F*_(1,39)_ = 0.15, *P* > 0.05]. The *post hoc* analysis revealed that both RRS (*P* < 0.05) and CVS (*P* < 0.05) reduced G, P_2_, and HR range, and these effects were inhibited by losartan treatment (*P* > 0.05).

**Table 1:** Parameters derived from non-linear (G, P_1_, P_2_, HR range, G, and BP_50_) and linear (slope bradycardia and slope tachycardia) regression analysis of the baroreflex in animals treated with vehicle or losartan and subjected to RRS or CVS.

Group	G (bpm/mmHg)	P_1_ (Δbpm)	P_2_ (Δbpm)	HR range (bpm)	BP_50_ (ΔmmHg)	Slope Bradycardia (bpm/mmHg)	Slope Tachycardia (bpm/mmHg)
**Vehicle**							
Control	-2.1 ± 0.2	-74 ± 5	134 ± 8	202 ± 6	-1.3 ± 5	-2.3 ± 0.2	-3.2 ± 0.5
RRS	-1.2 ± 0.2^∗^	-59 ± 6	94 ± 8^∗^	157 ± 5^∗^	-4.2 ± 4	-1.7 ± 0.2	-2.6 ± 0.4
CVS	-1.2 ± 0.2^∗^	-66 ± 6	96 ± 9^∗^	164 ± 12	-2.1 ± 3	-2.4 ± 0.3	-2.5 ± 0.5
**Losartan**							
Control	-2.3 ± 0.3	-78 ± 9	109 ± 12	187 ± 11	1.0 ± 3	-1.9 ± 0.3	-2.7 ± 0.6
RRS	-2.6 ± 0.2^#^	-89 ± 9	102 ± 11	199 ± 8^#^	0.0 ± 2	-2.6 ± 0.4	-2.0 ± 0.6
CVS	-1.8 ± 0.2^#^	-66 ± 9	115 ± 12	178 ± 18	-5.4 ± 2	-2.3 ± 0.3	-3.0 ± 0.5


**Values are mean ± SEM (*n* = 6: vehicle control, losartan control; *n* = 7: losartan CVS; *n* = 8: vehicle RRS, vehicle CVS, losartan RRS).**

**^∗^*P* < 0.05 versus control group within same condition, **^#^***P* < 0.05 versus respective group vehicle. Two-way ANOVA followed by Bonferroni’s *post hoc* test.**

The effect of MAP increase and decrease on the HR was also analyzed separately using linear regression analysis. However, the comparison of bradycardiac and tachycardiac response slopes did not indicate an effect of either stress [bradycardia: *F*_(2,39)_ = 0.38, *P* > 0.05; tachycardia: *F*_(2,39)_ = 0.78, *P* > 0.05] or treatment [bradycardia: *F*_(1,37)_ = 0.29, *P* > 0.05; tachycardia: *F*_(1,32)_ = 0.21, *P* > 0.05].

### Effects of Chronic Stress and Losartan Treatment on the Vascular Reactivity to Vasoactive Agents

**FIGURE 5:**
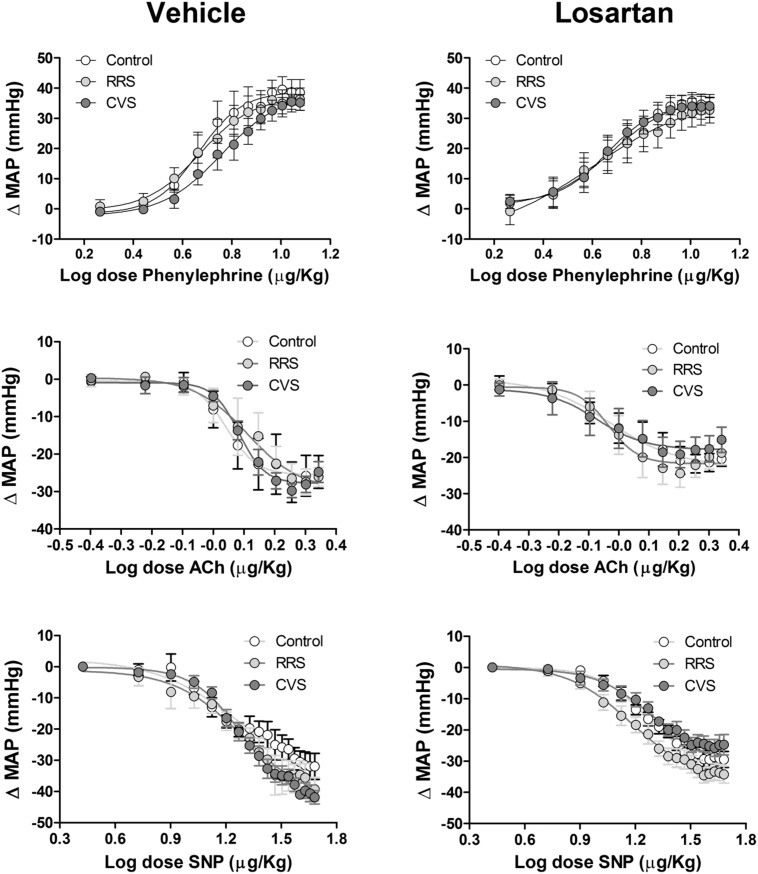
**Mean arterial pressure change (ΔMAP) evoked by vasoactive drugs in animals treated with vehicle or losartan control (white symbols) and subjected to RRS (light gray symbols) or CVS (dark gray symbols).** Increasing concentrations of phenylephrine **(Top)**, acetylcholine (Ach, **Middle**) and sodium nitroprusside (SNP, **Bottom**) in vehicle- (left) and losartan-treated animals. The circles represent the mean ± SEM. Non-linear regression analysis. (control vehicle: *n* = 6, control losartan: *n* = 8, RRS vehicle: *n* = 8, RRS losartan: *n* = 8, CVS vehicle: *n* = 8, CVS losartan: *n* = 7).

Results of the vascular reactivity to vasoactive agents are presented in **Figure 5** and **Table 2.**

**Table 2:** Maximal effect (*E*_max_) and dose at 50% of the MAP range (ED_50_) for phenylephrine (Phenyl), acetylcholine (Ach), and sodium nitroprusside (SNP) dose–response curves in animals treated with vehicle or losartan control and subjected to repeated restraint stress (RRS) or chronic variable stress (CVS).

Group	*n*	Phenyl ED_50_	*E*_max_	Ach ED_50_	*E*_max_	SNP ED_50_	*E*_max_
**Vehicle**							
Control	6	0.68 ± 0.05	38 ± 4	0.06 ± 0.06	-25 ± 4	1.19 ± 0.04	-32 ± 4
RRS	7	0.66 ± 0.07	36 ± 4	0.16 ± 0.03	-26 ± 2	1.26 ± 0.05	-39 ± 4
CVS	8	0.74 ± 0.04	35 ± 3	0.08 ± 0.02^$^	-25 ± 3	1.28 ± 0.03	-42 ± 2
**Losartan**							
Control	8	0.63 ± 0.07	33 ± 3	0.03 ± 0.02	-20 ± 2	1.22 ± 0.04	-29 ± 2
RRS	8	0.69 ± 0.07	33 ± 4	-0.01 ± 0.08^#^	-19 ± 3	1.18 ± 0.03	-34 ± 3
CVS	7	0.61 ± 0.05	34 ± 4	-0.02 ± 0.02	-15 ± 3^#^	1.18 ± 0.06	-25 ± 3^#$^


**Values are mean ± SEM.**

**^#^*P* < 0.05 vs. respective group losartan.**

**^$^*P* < 0.05 vs. respective RRS group. Two-way ANOVA followed by Bonferroni *post hoc* test.**

#### Phenylephrine

The intravenous infusion of the selective α_1_-adrenoceptor agonist phenylephrine dose-dependently evoked pressor responses in all groups. However, analysis of *E*_max_ and ED_50_ of the dose–response curves did not indicate an effect of either stress [*E*_max_: *F*_(2,39)_ = 0.11, *P* > 0.05; ED_50_: *F*_(2,39)_ = 0.08, *P* > 0.05] or treatment [*E*_max_: *F*_(1,39)_ = 1.16, *P* > 0.05; ED_50_: *F*_12,39)_ = 0.85, *P* > 0.05].

#### Acetylcholine

The systemic infusion of acetylcholine dose-dependently reduced the blood pressure in all groups. Comparison of the *E*_max_ and ED_50_ of the dose–response curves indicated an effect of treatment [*E*_max_: *F*_(1,39)_ = 8.21, *P* < 0.01; ED_50_: *F*_(1,39)_ = 5.11, *P* < 0.05]; however, stress [*F*_(2,39)_ = 0.48, *P* > 0.05; ED_50_: *F*_(2,37)_ = 0.25, *P* > 0.05] and stress × treatment interaction [*F*_(2,39)_ = 0.39, *P* > 0.05; ED_50_: *F*_(2,37)_ = 1.09, *P* > 0.05] had no effect. Nevertheless, the *post hoc* analysis did not reveal significant differences between the experimental groups.

#### Sodium Nitroprusside

The intravenous administration of the nitric oxide donor SNP dose-dependently reduced the blood pressure in all groups. Comparison of the *E*_max_ obtained from the dose–response curves indicated an effect of treatment [*F*_(1,39)_ = 10.83, *P* < 0.01] and stress × losartan interaction [*F*_(2,39)_ = 3.29, *P* < 0.05], without an effect of stress [*F*_(2,39)_ = 1.99, *P* > 0.05]. Analysis of the ED_50_ values did not indicate an effect of either stress [*F*_(2,39)_ = 0.12, *P* > 0.05], losartan [*F*_(1,39)_ = 2.29, *P* > 0.05], or stress × losartan interaction [*F*_(2,39)_ = 1.03, *p* > 0.05]. However, the *post hoc* analysis did not reveal significant differences between the experimental groups.

## Discussion

This is the first study to investigate the involvement of Ang II acting on the AT_1_ receptors in the autonomic and cardiovascular changes evoked by homotypic and heterotypic chronic stress regimens. **Table 3** summarizes the main findings reported in the present study.

**Table 3:** Summary of the effects of RRS and CVS in animals treated with vehicle or losartan.

		Vehicle		Losartan	
			
		RRS	CVS	RRS	CVS
**Somatic parameters**					
	Body weight	**–**	**↓**	**–**	**↓**
	Heart weight	**↑**	**↑**	**–**	**–**
	Adrenal weight	**–**	**–**	**–**	**–**
	Thymus weight	**–**	**–**	**–**	**–**
**HPA axis**					
	Corticosterone	**–**	**↑**	**–**	**↑**
**Cardiovascular baseline**					
	Mean Arterial pressure	**–**	**–**	**–**	**–**
	Diastolic Arterial pressure	**–**	**–**	**–**	**–**
	Systolic Arterial pressure	**–**	**–**	**↓**	**↓**
	HR	**–**	**–**	**–**	**–**
**Autonomic activity**					
	Cardiac sympathetic activity	**↑**	**–**	**–**	**↑**
	Cardiac Vagal activity	**↓**	**↓**	**–**	**–**
	Vascular sympathetic activity	**–**	**–**	**–**	**–**
	Intrinsic HR	**↓^∗^**	**↓^∗^**	**–**	**–**
	Baroreflex bradycardia	**–**	**–**	**–**	**–**
	Baroreflex tachycardia	**↓**	**↓**	**–**	**–**
**Vascular function**					
	Pressor response to phenylephrine	**–**	**–**	**–**	**–**
	Depressor effect of acetylcholine	**–**		**–**	**–**
	Depressor effect of Sodium nitroprusside	**–**	**–**	**–**	**–**


**Up or down arrows indicate significant increase or decrease, respectively.**

**^∗^Statistically non-significant but relevant (decrease of ∼10%).**

The findings that the body weight and plasma corticosterone concentration were uniquely affected by CVS is in line with previous evidence that changes in the somatic parameters and increased circulating glucocorticoid levels are more often observed following exposure to heterotypic than homotypic stressors (Magarinos and McEwen, 1995; Marin et al., 2007). Significant habituation of the HPA axis occurs over the course of repeated exposures to the same stressor, which minimizes the impact of the homotypic stressors on the basal plasma corticosterone concentration and somatic parameters (Herman, 2013). Interestingly, losartan treatment did not reverse the CVS-evoked corticosterone hypersecretion and reduction in body weight gain. In fact, the reduction in body weight was detected earlier in the losartan-treated animals, indicating that the treatment accentuated the CVS effect. Uresin et al. (2004) reported a small increase in the plasma corticosterone levels in rats subjected to RRS protocol, which was inhibited by systemic treatment with losartan. Taken together with the present findings, these results indicates a stress type-specific role of AT_1_ receptor on the impact of chronic stress in the HPA axis. Regarding the body weight, our findings are in line with a recent study that did not identify an effect of the systemic treatment with the AT_1_ receptor antagonist telmisartan on RRS-evoked body weight reduction (Wincewicz and Braszko, 2014). Nevertheless, the present findings provide the first evidence of a possible facilitatory role for the treatment with AT_1_ receptor antagonists on the effects of chronic stress regarding the body weight.

It has been demonstrated that AT_1_ receptor antagonists may reduce the body weight gain through activation of peroxisome proliferator-activated receptors (PPAR; He et al., 2010). Indeed, chronic treatment with losartan results in circulating levels of the metabolite EXP3179 that is sufficient for the activation of the PPARγ (Kappert et al., 2009). However, PPARγ plays a role in adipocyte differentiation and adipogenesis (Vidal-Puig et al., 1997), and the increased expression of this receptor has been implicated in obesity (Vidal-Puig et al., 1997). Therefore, the facilitatory effect of losartan on the CVS-evoked body weight reduction seems to be mediated by mechanisms other than PPARγ activation, and thus requires further investigation.

Neither RRS nor CVS affected the basal values of the arterial pressure and HR. Inconsistent findings have been reported regarding the impact of animal models of chronic stress on the arterial pressure (Nalivaiko, 2011; Crestani, 2016). In this sense, our results corroborate the evidence that chronic emotional stress does not affect the basal arterial pressure (Bechtold et al., 2009). Previous results have demonstrated that RRS does not affect the basal HR values (McDougall et al., 2000; Bechtold et al., 2009; Daubert et al., 2012; Duarte et al., 2015), whereas resting tachycardia has been reported in some studies following exposure to CVS (Grippo et al., 2002; Bouzinova et al., 2012; Duarte et al., 2015). Nevertheless, our results are in line with previous evidence that CVS does not affect HR (Cudnoch-Jedrzejewska et al., 2010; Flak et al., 2011; Xie et al., 2012; Almeida et al., 2015). It has been demonstrated that chronic treatment with losartan at doses ranging from 10 to 40 mg/kg decreased the basal arterial pressure in normotensive rats (Sacerdote et al., 1995; Bezerra et al., 2001; Leenen et al., 2001; Xavier et al., 2004; Koprdova et al., 2009). This is in accordance with our results.

Although there was no changes in the basal HR, a shift in the cardiac sympathovagal balance toward the sympathetic predominance was observed following exposure to either RRS or CVS. A tendency of a reduction in iHR (∼10%) was observed in animals subjected to both stressors, which suggests that a decrease in the cardiac pacemaker activity might have buffered the autonomic imbalance and avoided the emergence of changes in the resting HR. Treatment with losartan inhibited the alterations evoked by both RRS and CVS in the autonomic activity, which indicates a role of Ang II/AT_1_ receptors in these responses. Changes in the cardiac autonomic balance toward sympathetic predominance have been considered an important risk factor for cardiovascular morbidity and mortality (Carnevali and Sgoifo, 2014). Therefore, the present findings provide evidence that inhibition of the changes in the sympathetic and parasympathetic activities is an important mechanism that accounts for the protective effect of treatment with AT_1_ receptor antagonists against stress-evoked cardiovascular dysfunctions.

The classical pharmacological analysis indicated a reduction in the baroreflex activity in animals subjected to either RRS or CVS, which is in line with previous findings (Porter et al., 2004; Grippo et al., 2008; Almeida et al., 2015; Duarte et al., 2015). The sequence analysis technique did not reveal any effect of chronic stressors in the baroreflex. The present results corroborate recent evidence that chronic stress differently affect the baroreflex responses generated during spontaneous and full-range of arterial pressure changes (Almeida et al., 2015). Acute ablation of specific central nervous system regions has different effects on the baroreflex responses as assessed by the classical pharmacological approach and the sequence analysis technique (Crestani et al., 2010a; de Andrade et al., 2014), which indicates the presence of specific baroreflex circuitries generating reflex responses during spontaneous and evoked arterial pressure changes. Therefore, our findings indicate a selective influence of chronic stress on the baroreflex pathways involved in the responses generated during the full-range of arterial pressure changes. Impairment of the baroreflex activity is associated with an overactivity of the sympathetic tone and reduction of the cardiac parasympathetic activity (Grassi et al., 2004). Therefore, impairment of the baroreflex function may play a key role in the autonomic imbalance evoked by RRS and CVS.

Treatment with losartan inhibited the stress-evoked changes in baroreflex function. This finding indicates the involvement of Ang II/AT_1_ receptors in the baroreflex changes evoked by chronic stress, and reinforces the evidence of an involvement of the Ang II/AT_1_ receptors in the autonomic changes evoked by chronic stress. Chronic stress increases Ang II level in the plasma and brain tissue and the expression of AT_1_ receptors in the brain regions controlling the autonomic activity (Castren and Saavedra, 1988; Yang et al., 1994; McDougall et al., 2000). Activation of AT_1_ receptors in the brain elicits a set of changes in the autonomic activity, including an increase in the sympathetic activity, a decrease in the parasympathetic activity, and inhibition of the baroreflex function (Lindpaintner and Ganten, 1991; Head and Mayorov, 2006). Additionally, Ang II facilitates noradrenaline release from the cardiac sympathetic nerve terminals (Lindpaintner and Ganten, 1991). Therefore, inhibition of the stress-evoked sympathovagal imbalance by losartan treatment may be mediated via blockade of either the central or the peripheral AT_1_ receptors. Nevertheless, it has been proposed that modulation of the baroreflex function is mainly mediated via the action of Ang II in the brain (Averill and Diz, 2000; Head and Mayorov, 2006). This indicates that the effect of losartan on the baroreflex changes evoked by the chronic stressors is more likely related to the blockade of AT_1_ receptors in the brain baroreflex circuitry.

Previous studies using binding autoradiography consistently reported that peripheral administration of losartan at doses ranging from 1 to 100 mg/kg dose-dependently blocked the AT_1_ receptors in brain areas outside (e.g., the circumventricular organs) and within the blood–brain barrier (Song et al., 1991; Zhuo et al., 1994; Wang et al., 2003). These findings are further supported by functional evidence that losartan peripherally inhibited the pressor response, water intake, and vasopressin release in the circulation evoked by intracerebroventricular administration of Ang II (Polidori et al., 1996; Culman et al., 1999). Additionally, losartan may displays central effects in case of prolonged treatment or pathological conditions (Duron and Hanon, 2010; Karnik et al., 2015). Indeed, stress and hypertension are pathological conditions that can promote an increase in blood–brain barrier permeability (Skultétyová et al., 1998; Ueno et al., 2004), thus facilitating the action of losartan in the brain. These pieces of evidence support the hypothesis that the effects of losartan reported in the present study may be mediated by either peripheral or central blockade of the AT_1_ receptors.

Hypertension is associated with vascular dysfunction (Tang and Vanhoutte, 2010). Therefore, the similar response to the vasoactive agents in the stressed and non-stressed animals is in line with the lack of effects of RRS and CVS on the basal arterial pressure. The similar vasomotor sympathetic tone among the experimental groups (evidenced by analysis of oscillations of SAP at LF range) further supports the lack of changes in the arterial pressure. However, our findings were opposite to those of *in vitro* and *in vivo* studies reporting changes in the vascular reactivity to vasodilator and vasoconstrictor agents following exposure to either RRS or CVS (Neves et al., 2009; Isingrini et al., 2012; Baptista et al., 2014; Almeida et al., 2015; Duarte et al., 2015). The reasons for this discrepancy are not completely clear. Nevertheless, in the present study, the rats were handled daily for treatment with losartan. It has been reported that excessive handling by the experimenter, such as daily drug injection may buffer the effects of stress (Fone and Porkess, 2008). Moreover, chronic treatment with losartan did not evoke any change in the blood pressure response to vasodilator and vasoconstrictor agents, corroborating previous evidence that losartan treatment does not affect the vascular reactivity to vasoactive agents (Failli et al., 2009).

In summary, the present findings provide evidence of the involvement of Ang II/AT_1_ receptors in the cardiac sympathovagal imbalance and the changes in the baroreflex function evoked by both homotypic and heterotypic chronic stress regimens. Results of the present study also provide evidence that the increased circulating corticosterone level evoked by CVS is independent of Ang II/AT_1_ receptors, whereas the reduction in the body weight gain evoked by heterotypic stressors may be facilitated by treatment with losartan.

## Author Contributions

WC-F and CC contributed to the conception and design of the work. WC-F, JV, JA, and LGS contributed to the acquisition, analysis, and interpretation of data. WC-F and CC drafted the work. JV, JA, and LGS critically revised the manuscript and CC approved the final version to be published.

## Conflict of Interest Statement

The authors declare that the research was conducted in the absence of any commercial or financial relationships that could be construed as a potential conflict of interest.

## Footnotes

1 https://www.sites.google.com/site/cardioseries/home

## References

[B1] Almeida J. Duarte J. O. Oliveira L. A. Crestani C. C. (2015). Effects of nitric oxide synthesis inhibitor or fluoxetine treatment on depression-like state and cardiovascular changes induced by chronic variable stress in rats. *Stress* 18 462–474. 10.3109/10253890.2015.1038993 26068517

[B2] Averill D. B. Diz D. I. (2000). Angiotensin peptides and baroreflex control of sympathetic outflow: pathways and mechanisms of the medulla oblongata. *Brain Res. Bull.* 51 119–128. 10.1016/S0361-9230(99)00237-3 10709957

[B3] Bali A. Jaggi A. S. (2013). Angiotensin as stress mediator: role of its receptor and interrelationships among other stress mediators and receptors. *Pharmacol. Res.* 76 49–57. 10.1016/j.phrs.2013.07.004 23892268

[B4] Baptista R. de F. F. Taipeiro E. de F. Queiroz R. H. C. Chies A. B. (2014). Stress alone or associated with ethanol induces prostanoid release in rat aorta via α2-adrenoceptor. *Arq. Bras. Cardiol.* 102 211–218. 10.5935/abc.20140015 24676223 PMC3987321

[B5] Bechtold A. G. Patel G. Hochhaus G. Scheuer D. A. (2009). Chronic blockade of hindbrain glucocorticoid receptors reduces blood pressure responses to novel stress and attenuates adaptation to repeated stress. *Am. J. Physiol. Regul. Integr. Comp. Physiol.* 296 R1445–R1454. 10.1152/ajpregu.00095.2008 19279295 PMC2689825

[B6] Bezerra S. M. M. S. dos Santos C. M. Moreira E. D. Krieger E. M. Michelini L. C. (2001). Chronic AT 1 receptor blockade alters autonomic balance and sympathetic responses in hypertension. *Hypertension* 38 569–575. 10.1161/hy09t1.095393 11566933

[B7] Bouzinova E. V. Møller-Nielsen N. Boedtkjer D. B. Broegger T. Wiborg O. Aalkjaer C. (2012). Chronic mild stress-induced depression-like symptoms in rats and abnormalities in catecholamine uptake in small arteries. *Psychosom. Med.* 74 278–287. 10.1097/PSY.0b013e31824c40a9 22408132

[B8] Busnardo C. Tavares R. F. Correa F. M. A. (2014). Angiotensinergic neurotransmission in the paraventricular nucleus of the hypothalamus modulates the pressor response to acute restraint stress in rats. *Neuroscience* 270 12–19. 10.1016/j.neuroscience.2014.03.064 24717718

[B9] Carnevali L. Sgoifo A. (2014). Vagal modulation of resting heart rate in rats: the role of stress, psychosocial factors, and physical exercise. *Front. Physiol.* 5:118 10.3389/fphys.2014.00118 PMC3970013 24715877

[B10] Castren E. Saavedra J. M. (1988). Repeated stress increases the density of angiotensin II binding sites in rat paraventricular nucleus and subfornical organ. *Endocrinology* 122 370–372. 10.1210/endo-122-1-370 3335214

[B11] Chung I.-M. Kim Y.-M. Yoo M.-H. Shin M.-K. Kim C.-K. Suh S. H. (2010). Immobilization stress induces endothelial dysfunction by oxidative stress via the activation of the angiotensin II/its type I receptor pathway. *Atherosclerosis* 213 109–114. 10.1016/j.atherosclerosis.2010.08.052 20850747

[B12] Conti L. H. Shannon M. H. Murry J. D. Printz M. P. (2001). Repeated restraint stress-induced increase in baroreceptor reflex sensitivity: role of corticotropin-releasing factor. *Neuropeptides* 35 71–81. 10.1054/npep.2001.0847 11384202

[B13] Crestani C. C. (2016). Emotional stress and cardiovascular complications in animal models: a review of the influence of stress type. *Front. Physiol.* 7:251 10.3389/fphys.2016.00251 PMC4919347 27445843

[B14] Crestani C. C. Alves F. H. F. Busnardo C. Resstel L. B. M. Correa F. M. A. (2010a). N-methyl-D-aspartate glutamate receptors in the hypothalamic paraventricular nucleus modulate cardiac component of the baroreflex in unanesthetized rats. *Neurosci. Res.* 67 317–326. 10.1016/j.neures.2010.05.001 20472007

[B15] Crestani C. C. Tavares R. F. Alves F. H. F. Resstel L. B. M. Correa F. M. A. (2010b). Effect of acute restraint stress on the tachycardiac and bradycardiac responses of the baroreflex in rats. *Stress* 13 61–72. 10.3109/10253890902927950 20105054

[B16] Crestani C. C. Tavares R. F. Guimarães F. S. Correa F. M. A. Joca S. R. L. Resstel L. B. M. (2011). Chronic fluoxetine treatment alters cardiovascular functions in unanesthetized rats. *Eur. J. Pharmacol.* 670 527–533. 10.1016/j.ejphar.2011.09.030 21963526

[B17] Cudnoch-Jedrzejewska A. Szczepanska-Sadowska E. Dobruch J. Gomolka R. Puchalska L. (2010). Brain vasopressin V(1) receptors contribute to enhanced cardiovascular responses to acute stress in chronically stressed rats and rats with myocardial infarcton. *Am. J. Physiol. Regul. Integr. Comp. Physiol.* 298 R672–R680. 10.1152/ajpregu.00543.2009 20042688

[B18] Culman J. von Heyer C. Piepenburg B. Rascher W. Unger T. (1999). Effects of systemic treatment with irbesartan and losartan on central responses to angiotensin II in conscious, normotensive rats. *Eur. J. Pharmacol.* 367 255–265. 10.1016/S0014-2999(98)00983-2 10079000

[B19] Daubert D. L. McCowan M. Erdos B. Scheuer D. A. (2012). Nucleus of the solitary tract catecholaminergic neurons modulate the cardiovascular response to psychological stress in rats. *J. Physiol.* 590 4881–4895. 10.1113/jphysiol.2012.232314 22753543 PMC3487043

[B20] de Andrade O. Borghi S. M. de Souza H. C. D. Fontes M. A. P. Martins-Pinge M. C. (2014). Paraventricular nucleus of hypothalamus participates in the sympathetic modulation and spontaneous fluctuation of baroreflex during head up tilt in unanesthetized rats. *Neurosci. Lett.* 558 1–7. 10.1016/j.neulet.2013.09.039 24176880

[B21] de Gasparo M. Catt K. J. Inagami T. Wright J. W. Unger T. (2000). International union of pharmacology. XXIII. The angiotensin II receptors. *Pharmacol. Rev.* 52 415–472. 10977869

[B22] Duarte J. O. Cruz F. C. Leão R. M. Planeta C. S. Crestani C. C. (2015). Stress vulnerability during adolescence. *Psychosom. Med.* 77 186–199. 10.1097/PSY.0000000000000141 25659080

[B23] Duron E. Hanon O. (2010). Antihypertensive treatments, cognitive decline, and dementia. *J. Alzheimers Dis.* 20 903–914. 10.3233/JAD-2010-091552 20182022

[B24] Erdos B. Cudykier I. Woods M. Basgut B. Whidden M. Tawil R. (2010). Hypertensive effects of central angiotensin II infusion and restraint stress are reduced with age. *J. Hypertens.* 28 1298–1306. 10.1097/HJH.0b013e328338a075 20308921

[B25] Failli P. Alfarano C. Franchi-Micheli S. Mannucci E. Cerbai E. Mugelli A. (2009). Losartan counteracts the hyper-reactivity to angiotensin II and ROCK1 over-activation in aortas isolated from streptozotocin-injected diabetic rats. *Cardiovasc. Diabetol.* 8 32 10.1186/1475-2840-8-32 PMC2711933 19545435

[B26] Flak J. N. Jankord R. Solomon M. B. Krause E. G. Herman J. P. (2011). Opposing effects of chronic stress and weight restriction on cardiovascular, neuroendocrine and metabolic function. *Physiol. Behav.* 104 228–234. 10.1016/j.physbeh.2011.03.002 21396386 PMC3395375

[B27] Flak J. N. Solomon M. B. Jankord R. Krause E. G. Herman J. P. (2012). Identification of chronic stress-activated regions reveals a potential recruited circuit in rat brain. *Eur. J. Neurosci.* 36 2547–2555. 10.1111/j.1460-9568.2012.08161.x 22789020 PMC4538599

[B28] Fone K. C. F. Porkess M. V. (2008). Behavioural and neurochemical effects of post-weaning social isolation in rodents-relevance to developmental neuropsychiatric disorders. *Neurosci. Biobehav. Rev.* 32 1087–1102. 10.1016/j.neubiorev.2008.03.003 18423591

[B29] Granjeiro É. M. Marroni S. S. Martins Dias D. P. Heck Bonagamba L. G. Costa K. M. dos Santos J. C. (2014). Behavioral and cardiorespiratory responses to bilateral microinjections of oxytocin into the central nucleus of amygdala of Wistar rats, an experimental model of compulsion. *PLoS ONE* 9:e99284 10.1371/journal.pone.0099284 PMC4103777 25036025

[B30] Grassi G. Seravalle G. Dell’Oro R. Facchini A. Ilardo V. Mancia G. (2004). Sympathetic and baroreflex function in hypertensive or heart failure patients with ventricular arrhythmias. *J. Hypertens.* 22 1747–1753. 10.1097/00004872-200409000-00019 15311103

[B31] Grippo A. J. Moffitt J. A. Johnson A. K. (2002). Cardiovascular alterations and autonomic imbalance in an experimental model of depression. *Am. J. Physiol.* 282 R1333–R1341. 10.1152/ajpregu.00614.2001 11959673

[B32] Grippo A. J. Moffitt J. A. Johnson A. K. (2008). Evaluation of baroreceptor reflex function in the chronic mild stress rodent model of depression. *Psychosom. Med.* 70 435–443. 10.1097/PSY.0b013e31816ff7dd 18480191 PMC3399455

[B33] Grippo A. J. Santos C. M. Johnson R. F. Beltz T. G. Martins J. B. Felder R. B. (2004). Increased susceptibility to ventricular arrhythmias in a rodent model of experimental depression. *Am. J. Physiol. Heart Circ. Physiol.* 286 H619–H626. 10.1152/ajpheart.00450.2003 14715499

[B34] Haile C. N. GrandPre T. Kosten T. A. (2001). Chronic unpredictable stress, but not chronic predictable stress, enhances the sensitivity to the behavioral effects of cocaine in rats. *Psychopharmacology (Berl.)* 154 213–220. 10.1007/s002130000650 11314684

[B35] He H. Yang D. Ma L. Luo Z. Ma S. Feng X. (2010). Telmisartan prevents weight gain and obesity through activation of peroxisome proliferator-activated receptor-delta-dependent pathways. *Hypertension* 55 869–879. 10.1161/HYPERTENSIONAHA.109.143958 20176998

[B36] Head G. A. (1996). Proceedings of the symposium “angiotensin AT1 receptors: from molecular physiology to therapeutics”: ROLE OF AT1 RECEPTORS IN THE CENTRAL CONTROL OF SYMPATHETIC VASOMOTOR FUNCTION. *Clin. Exp. Pharmacol. Physiol.* 23 93–98. 10.1111/j.1440-1681.1996.tb02820.x 21143280

[B37] Head G. A. Mayorov D. N. (2006). Central angiotensin and baroreceptor control of circulation. *Ann. N. Y. Acad. Sci.* 940 361–379. 10.1111/j.1749-6632.2001.tb03691.x 11458693

[B38] Head G. A. McCarty R. (1987). Vagal and sympathetic components of the heart rate range and gain of the baroreceptor-heart rate reflex in conscious rats. *J. Auton. Nerv. Syst.* 21 203–213. 10.1016/0165-1838(87)90023-3 3450695

[B39] Herman J. P. (2013). Neural control of chronic stress adaptation. *Front. Behav. Neurosci.* 7:61 10.3389/fnbeh.2013.00061 PMC3737713 23964212

[B40] Isingrini E. Belzung C. Freslon J.-L. Machet M.-C. Camus V. (2012). Fluoxetine effect on aortic nitric oxide-dependent vasorelaxation in the unpredictable chronic mild stress model of depression in mice. *Psychosom. Med.* 74 63–72. 10.1097/PSY.0b013e31823a43e0 22210237

[B41] Janssen B. J. Leenders P. J. Smits J. F. (2000). Short-term and long-term blood pressure and heart rate variability in the mouse. *Am. J. Physiol. Regul. Integr. Comp. Physiol.* 278 R215–R225. 10644642 10.1152/ajpregu.2000.278.1.R215

[B42] Jarczok M. N. Jarczok M. Mauss D. Koenig J. Li J. Herr R. M. (2013). Autonomic nervous system activity and workplace stressors–a systematic review. *Neurosci. Biobehav. Rev.* 37 1810–1823. 10.1016/j.neubiorev.2013.07.004 23891906

[B43] Jezova D. Ochedalski T. Kiss A. Aguilera G. (1998). Brain angiotensin II modulates sympathoadrenal and hypothalamic pituitary adrenocortical activation during stress. *J. Neuroendocrinol.* 10 67–72. 10.1046/j.1365-2826.1998.00182.x 9510060

[B44] Just A. Faulhaber J. Ehmke H. (2000). Autonomic cardiovascular control in conscious mice. *Am. J. Physiol. Regul. Integr. Comp. Physiol.* 279 R2214–R2221. 11080088 10.1152/ajpregu.2000.279.6.R2214

[B45] Kappert K. Tsuprykov O. Kaufmann J. Fritzsche J. Ott I. Goebel M. (2009). Chronic treatment with losartan results in sufficient serum levels of the metabolite EXP3179 for PPAR activation. *Hypertension* 54 738–743. 10.1161/HYPERTENSIONAHA.109.132886 19687349

[B46] Karnik S. S. Unal H. Kemp J. R. Tirupula K. C. Eguchi S. Vanderheyden P. M. L. (2015). Angiotensin receptors: interpreters of pathophysiological angiotensinergic stimuli. *Pharmacol. Rev.* 67 754–819. 10.1124/pr.114.010454 26315714 PMC4630565

[B47] Kivimäki M. Virtanen M. Elovainio M. Kouvonen A. Väänänen A. Vahtera J. (2006). Work stress in the etiology of coronary heart disease—a meta-analysis. *Scand. J. Work Environ. Health* 32 431–442. 10.5271/sjweh.1049 17173200

[B48] Kopp B. L. Wick D. Herman J. P. (2013). Differential effects of homotypic vs. heterotypic chronic stress regimens on microglial activation in the prefrontal cortex. *Physiol. Behav.* 122 246–252. 10.1016/j.physbeh.2013.05.030 23707717 PMC3797875

[B49] Koprdova R. Cebova M. Kristek F. (2009). Long-term effect of losartan administration on blood pressure, heart and structure of coronary artery of young spontaneously hypertensive rats. *Physiol. Res.* 58 327–335. 18637711 10.33549/physiolres.931528

[B50] Kubo T. Numakura H. Endo S. Hagiwara Y. Fukumori R. (2001). Angiotensin receptor blockade in the anterior hypothalamic area inhibits stress-induced pressor responses in rats. *Brain Res. Bull.* 56 569–574. 10.1016/S0361-9230(01)00729-8 11786243

[B51] Leenen F. H. White R. Yuan B. (2001). Isoproterenol-induced cardiac hypertrophy: role of circulatory versus cardiac renin-angiotensin system. *Am. J. Physiol. Heart Circ. Physiol.* 281 H2410–H2416. 11709406 10.1152/ajpheart.2001.281.6.H2410

[B52] Lindpaintner K. Ganten D. (1991). The cardiac renin-angiotensin system. An appraisal of present experimental and clinical evidence. *Circ. Res.* 68 905–921. 10.1161/01.RES.68.4.905 2009615

[B53] Magarinos A. M. McEwen B. S. (1995). Stress-induced atrophy of apical dendrites of hippocampal CA3c neurons: comparison of stressors. *Neuroscience* 69 83–88. 10.1016/0306-4522(95)00256-I 8637635

[B54] Malliani A. Pagani M. Lombardi F. Cerutti S. (1991). Cardiovascular neural regulation explored in the frequency domain. *Circulation* 84 482–492. 10.1161/01.CIR.84.2.482 1860193

[B55] Marin M. T. Cruz F. C. Planeta C. S. (2007). Chronic restraint or variable stresses differently affect the behavior, corticosterone secretion and body weight in rats. *Physiol. Behav.* 90 29–35. 10.1016/j.physbeh.2006.08.021 17023009

[B56] McCarty R. (2016). Learning about stress: neural, endocrine and behavioral adaptations. *Stress* 10.1080/10253890.2016.1192120 [Epub ahead of print]. 27294884

[B57] McDougall S. J. Roulston C. A. Widdop R. E. Lawrence A. J. (2000). Characterisation of vasopressin V1A, angiotensin AT1 and AT2 receptor distribution and density in normotensive and hypertensive rat brain stem and kidney: effects of restraint stress11Published on the World Wide Web on 2 October 2000. *Brain Res.* 883 148–156. 10.1016/S0006-8993(00)02917-6 11063999

[B58] McKinley M. J. Albiston A. L. Allen A. M. Mathai M. L. May C. N. McAllen R. M. (2003). The brain renin–angiotensin system: location and physiological roles. *Int. J. Biochem. Cell Biol.* 35 901–918. 10.1016/S1357-2725(02)00306-0 12676175

[B59] Ménard J. (1993). Anthology of the renin-angiotensin system: a one hundred reference approach to angiotensin II antagonists. *J. Hypertens. Suppl.* 11 S3–S11. 8315517

[B60] Nalivaiko E. (2011). Animal models of psychogenic cardiovascular disorders: what we can learn from them and what we cannot. *Clin. Exp. Pharmacol. Physiol.* 38 115–125. 10.1111/j.1440-1681.2010.05465.x 21105893

[B61] Neves V. J. Moura M. J. C. S. Tamascia M. L. Ferreira R. Silva N. S. Costa R. (2009). Proatherosclerotic effects of chronic stress in male rats: altered phenylephrine sensitivity and nitric oxide synthase activity of aorta and circulating lipids. *Stress* 12 320–327. 10.1080/10253890802437779 19085621

[B62] Pastor-Ciurana J. Rabasa C. Ortega-Sánchez J. A. Sanchís-Ollè M. Gabriel-Salazar M. Ginesta M. (2014). Prior exposure to repeated immobilization or chronic unpredictable stress protects from some negative sequels of an acute immobilization. *Behav. Brain Res.* 265 155–162. 10.1016/j.bbr.2014.02.028 24583189

[B63] Polidori C. Ciccocioppo R. Pompei P. Cirillo R. Massi M. (1996). Functional evidence for the ability of angiotensin AT1 receptor antagonists to cross the blood-brain barrier in rats. *Eur. J. Pharmacol.* 307 259–267. 10.1016/0014-2999(96)00270-1 8836613

[B64] Porter J. P. Phillips A. Rich J. Wright D. (2004). Effect of chronic stress on the cardiac baroreflex in the post-weanling rat. *Life Sci.* 75 1595–1607. 10.1016/j.lfs.2004.03.018 15261764

[B65] Ramaekers D. Beckers F. Demeulemeester H. Aubert A. E. (2002). Cardiovascular autonomic function in conscious rats: a novel approach to facilitate stationary conditions. *Ann. Noninvasive Electrocardiol.* 7 307–318. 10.1111/j.1542-474X.2002.tb00179.x 12431308 PMC7027617

[B66] Sacerdote A. Cosenzi A. Bocin E. Molino R. Seculin P. Plazzotta N. (1995). Effects of chronic treatment with losartan on blood pressure, endothelin-like immunoreactivity and nitric oxide in normotensive rats. *J. Hypertens.* 13 1670–1673. 10.1097/00004872-199512010-00029 8903630

[B67] Saiki Y. Watanabe T. Tan N. Matsuzaki M. Nakamura S. (1997). Role of central ANG II receptors in stress-induced cardiovascular and hyperthermic responses in rats. *Am. J. Physiol.* 272 R26–R33. 9038987 10.1152/ajpregu.1997.272.1.R26

[B68] Sarnyai Z. Bíró E. Penke B. Telegdy G. (1992). The cocaine-induced elevation of plasma corticosterone is mediated by endogenous corticotropin-releasing factor (CRF) in rats. *Brain Res.* 589 154–156. 10.1016/0006-8993(92)91176-F 1330207

[B69] Skultétyová I. Tokarev D. Jezová D. (1998). Stress-induced increase in blood-brain barrier permeability in control and monosodium glutamate-treated rats. *Brain Res. Bull.* 45 175–178. 10.1016/S0361-9230(97)00335-3 9443836

[B70] Smith B. L. Schmeltzer S. N. Packard B. A. Sah R. Herman J. P. (2016). Divergent effects of repeated restraint versus chronic variable stress on prefrontal cortical immune status after LPS injection. *Brain. Behav. Immun.* 10.1016/j.bbi.2016.05.004 [Epub ahead of print]. PMC5015433 27177449

[B71] Song K. F. Zhuo J. L. Mendelsohn F. A. (1991). Access of peripherally administered DuP 753 to rat brain angiotensin II receptors. *Br. J. Pharmacol.* 104 771–772. 10.1111/j.1476-5381.1991.tb12503.x 1810594 PMC1908825

[B72] Tang E. H. C. Vanhoutte P. M. (2010). Endothelial dysfunction: a strategic target in the treatment of hypertension? *Pfluügers Arch.* 459 995–1004. 10.1007/s00424-010-0786-4 20127126

[B73] Ueno M. Sakamoto H Liao Y. J. Onodera M. Huang C. L. Miyanaka H. (2004). Blood-brain barrier disruption in the hypothalamus of young adult spontaneously hypertensive rats. *Histochem. Cell Biol.* 122 131–137. 10.1007/s00418-004-0684-y 15258771

[B74] Uresin Y. Erbas B. Ozek M. Ozkök E. Gürol A. O. (2004). Losartan may prevent the elevation of plasma glucose, corticosterone and catecholamine levels induced by chronic stress. *J. Renin Angiotensin Aldosterone Syst.* 5 93–96. 10.3317/jraas.2004.017 15295722

[B75] Vidal-Puig A. J. Considine R. V. Jimenez-Liñan M. Werman A. Pories W. J. Caro J. F. (1997). Peroxisome proliferator-activated receptor gene expression in human tissues. Effects of obesity, weight loss, and regulation by insulin and glucocorticoids. *J. Clin. Invest.* 99 2416–2422. 10.1172/JCI119424 9153284 PMC508081

[B76] Wang J. M. Tan J. Leenen F. H. H. (2003). Central nervous system blockade by peripheral administration of AT1 receptor blockers. *J. Cardiovasc. Pharmacol.* 41 593–599. 10.1097/00005344-200304000-00012 12658061

[B77] Wincewicz D. Braszko J. J. (2014). Telmisartan attenuates cognitive impairment caused by chronic stress in rats. *Pharmacol. Rep.* 66 436–441. 10.1016/j.pharep.2013.11.002 24905520

[B78] Wright J. W. Harding J. W. (2011). Brain renin-angiotensin—A new look at an old system. *Prog. Neurobiol.* 95 49–67. 10.1016/j.pneurobio.2011.07.001 21777652

[B79] Xavier F. E. Yogi Á Callera G. E. Tostes R. C. Alvarez Y. Salaices M. (2004). Contribution of the endothelin and renin-angiotensin systems to the vascular changes in rats chronically treated with ouabain. *Br. J. Pharmacol.* 143 794–802. 10.1038/sj.bjp.0705994 15477225 PMC1575934

[B80] Xie F. Sun L. Su X. Wang Y. Liu J. Zhang R. (2012). Neuropeptide Y reverses chronic stress-induced baroreflex hypersensitivity in rats. *Cell. Physiol. Biochem.* 29 463–474. 10.1159/000338500 22508053

[B81] Yang G. Xi Z.-X. Wan Y. Wang H. Bi G. (1994). Changes in circulating and tissue angiotensin II during acute and chronic stress. *Neurosignals* 2 166–172. 10.1159/000109488 8004155

[B82] Zhuo J. Song K. Abdelrahman A. Mendelsohn F. A. O. (1994). Blockade by intravenous losartan of at 1 angiotensin ii receptors in rat brain, kidney and adrenals demonstrated by in vitro autoradiography. *Clin. Exp. Pharmacol. Physiol.* 21 557–567. 10.1111/j.1440-1681.1994.tb02555.x 7982288

